# Dispensary of Jefferson Medical College

**Published:** 1844-07-13

**Authors:** Edward R. Squibb


### CLINICAL LECTURES AND REPORTS.

### DISPENSARY OF JEFFERSON MEDICAL COLLEGE.

PROFESSOR BACHE'S CLINIC (MEDICAL.)

[Reported by Edward R. Squibb.]

*Wednesday, May 1st*, 1844.

CASE I.—Joseph J., æt. 20. Complains of stiffness and painful sensation in the breast; rigidity of the jaws, not being able to open them more than half an inch; and spasmodic contractions of the diaphragm, interfering with breathing and speaking.

The history of the case, as elicited by questions put to the patient, is as follows:—About four weeks since, whilst walling a well, he accidentally struck his finger with a hatchet, bruising it very much without breaking the skin. Observing that the blood collected under the nail, he made an opening, through which it escaped; after which he experienced little inconvenience from the accident. At the commencement of this week, and three weeks after the occurrence, he first perceived a difficulty in moving his jaws. This attracted little attention, until by its increase, and the supervention of like stiffness in the chest, with slight pain on speaking, the patient found himself incapable of making any exertion; and now, at the end of the fourth day from the commencement of the present train of symptoms, is unable to eat solid food. He now has spasmodic impediment in his speech; involuntary twitching of the head; and increased pain in speaking. The affection seems confined entirely to the chest and jaws; no difficulty experienced in swallowing; bowels regular; appetite good; pulse natural; rest good; and appears free from pain except when speaking or making other muscular exertion; has had a mustard plaster applied to his breast, and a ginger poultice to his jaws, but without any relief.

In view of the symptoms of this case, it must be very apparent that there exists some source of nervous irritation in the system, which gives rise to these irregularities of muscular action.

In searching for the cause in the history of which the patient has given us, our attention is naturally drawn to the accident which he has mentioned, especially when we remember that tetanus sometimes follows wounds and contusions of a very trivial character.

Ordered to soak the finger well in warm water, and apply a laudanum poultice to it; and take a pill consisting of ½ grain of extract of belladonna with three grains of Dover's powder every three hours; the patient to be closely watched, and any increase of symptoms reported.

Case II.—Thomas A., æt. 34. This case has been under treatment for three weeks, for incipient phthisis; has had three grains of iodide of potassium three times a day as an alterative; a plaster of pitch with cantharides over the seat of pain; and as an anodyne, the solution of sulphate of morphia at bed time. To-day the patient feels about the same; has rested better and expectorated more freely through the week. On percussion the chest sounds better than might be expected, though dull under the left clavicle. Pulse 128; tongue red at the tip, and white behind; expectoration more free, and of a thicker consistence.

Ordered to continue the treatment.

Case III.—Mrs. S., æt. 40. Has been under treatment three weeks, for ascites with tendency to anasarca. Was ordered to take powders of crem. tartar and jalap, under the use of which she is improving. Complains to-day of sore throat and loss of appetite.

Ordered to continue the powders, and to rub the throat with liniment of ammonia, using at the same time a gargle of sage tea, borax and honey.

Case IV.—Margaret C., æt. 38. Complains of shortness of breath; burning pain between the shoulders; loss of appetite; great debility; and a feeling of oppression or constriction of the chest; tongue natural; pulse 64; catamenia irregular since January; skin moist; countenance pale and anxious.

Ordered a purge of 2 comp. cathart. pills every night, and the following powders, one to be taken three times a day:

℞. Pulv. Colombæ,

" Zinzib. *aa* ℥ij.

Ferri subcarb. ℥j.

M. ft. chart. xij.

Case V.—Hugh D., æt. 31. Has intermittent fever; chills severe; appetite poor; bowels irregular; pulse good; tongue natural.

Ordered an emetic of ipecac. ℈j., tart. antim. gr. j. to be taken immediately, followed by a pill of sulphate of quinia every two hours, during the intermissions.

Case VI.—James C., æt. 26. Has been under treatment several weeks for pain in the lumbar region (see former report); has felt somewhat better through the week, until this morning, when he was seized with cramp, since which the pain has increased.

Ordered to take pills consisting of ¼ grain of ext. of belladonna and 3 grains of Dover's powder, one morning and evening.

Case VII.—Ellen W. Complains of swelling of the abdomen, which commenced nine weeks ago, immediately after confinement. Urine deficient and high coloured; stools whitish and regular; appetite tolerable; pulse good; tongue natural, and rest good.

Ordered to take a pill consisting of ½ grain of calomel and 3 grains of powdered squills, 3 times a day.

Case VIII.—Jane McA., æt. 31. Has colliquative diarrhœa. Pulse small; tongue inflamed; countenance livid, with an anxious expression; complains of great debility; loss of appetite, and distressing tenesmus, with prolapsus ani.

Ordered to take ½ grain of opium, with 1½ grains of ipecac. in form of pill after each stool, and a milk diet—as milk punch, milk thickened with boiled flour, &c., and to wean the child which she is at present nursing.

